# Impact of environmental education on environmental quality under the background of low-carbon economy

**DOI:** 10.3389/fpubh.2023.1128791

**Published:** 2023-02-16

**Authors:** Ye Wu, Jiawei Wan, Wen Yu

**Affiliations:** ^1^School of Business Administration and Customs Affairs, Shanghai Customs College, Shanghai, China; ^2^Faculty of Business Economics, Shanghai Business School, Shanghai, China; ^3^School of Humanities, Shanghai University of Finance and Economics, Shanghai, China

**Keywords:** low-carbon economy, environmental health education, pollution control, household consumption, environmental quality

## Abstract

**Introduction:**

How does environmental education affect environmental quality? There is no consensus among theorists. This paper is devoted to exploring the influence mechanism of environmental education and environmental quality under the background of a low-carbon economy from a theoretical model and empirical analysis.

**Methods:**

The research method of this paper includes two aspects. First, from the consideration of the central planner, this paper draws on and improves the Ramsey Model to explore the interaction mechanism among environmental education, environmental quality and green growth. Second, this paper uses provincial panel data from China from 2011 to 2017 for empirical analysis, which mainly verifies the impact mechanism of environmental education on environmental quality.

**Results and discussion:**

The theoretical model shows that environmental education enhances green consumption intention through residents' environmental awareness and enhances enterprises' cleaner production motivation through environmental pressure. Correspondingly, the pressure to improve environmental quality will also promote the economy's endogenous growth through the digital economy's transformation and the accumulation of human capital. The empirical analysis confirms that environmental education can improve environmental quality through green consumption and pollution control. Still, the effect of improving environmental quality only through pollution control is not apparent, and pollution control needs to be combined with environmental education, especially in high-pollution areas. Finally, this paper puts forward some suggestions for optimizing environmental education.

## Introduction

Global climate change has become one of the most significant challenges to human development, dramatically promoting global political consensus and significant actions to address climate change. At the first World Summit on Sustainable Development (WSSD) in Johannesburg in 2002, delegates called for “encouraging and promoting the development of a 10-year framework for Sustainable Consumption and Production Plans (SCP) to support regional and national initiatives to accelerate the transition to SCP”. Promoting sustainable development through pollution control and green consumption is increasingly becoming the international community's consensus. When the level of economic development reaches a certain stage, the government should pay more attention to sustainable development in public life, education, and environmental protection (1). In recent years, more and more governments have also elevated “carbon neutrality” as a national strategy, committed to the vision of a carbon-free future through environmental education, pollution control, and green consumption. The European Union has taken the lead in announcing an absolute target to cut greenhouse gas emissions by at least 55% from 1990 levels by 2030 and make Europe the world's first “carbon neutral” continent by 2050. China has pledged to stop increasing its carbon dioxide emissions by 2030 and gradually reduce them after peaking, setting a strategic goal of achieving carbon neutrality by 2060. In 2021, the Opinions of the CPC Central Committee and the State Council on Fully, Accurately, and Comprehensively Implementing the New Development Concept to achieve carbon peak and neutrality began to be issued nationwide. The Opinions set a “timetable” and “road map” for achieving carbon peak and neutrality. It is a major measure for China to promote high-quality development, strengthen ecological civilization construction, safeguard national energy security and build a community with a shared future for humanity.

Economic growth is becoming more and more dependent on the natural environment. Scholars have paid more and more attention to the relationship between economic growth and sustainable development of the natural environment (2, 3). In the initial stage of economic growth, industrialization usually brings serious environmental pollution, such as air pollution, water and soil environmental pollution (4); this phenomenon also exists in China (5). With the improvement of the industrialization process, sustainable and high-quality development is the new theme of China's current economy and society. If we still blindly pursue economic growth rate, ignoring environmental carrying capacity and “trading pollution for GDP,” which ran counter not only to the concept of sustainable development but also the goal of high-quality economic development.

Existing studies found that environmental education affects environmental quality. On the one hand, environmental education increases the environmental awareness of residents or consumers, forcing local pollution control and environmental legislation to improve environmental quality (6). On the other hand, environmental education promoted green consumption (7) and thus reduced pollution control. However, some scholars believe that the impact of pollution control on environmental quality is uncertain (8, 9). Is pollution control the core channel through which environmental education affects environmental quality? There is no consensus among theorists. Therefore, this paper aims to theoretically analyze and empirically test the transmission path and influence mechanism of environmental education, green consumption, green production, pollution control, and other factors on environmental quality.

The rest of this paper is arranged as follows: The second part is a literature review and theoretical hypotheses. The third part is theoretical model analysis. The fourth part is empirical analysis. The conclusion and discussion are presented in the last section.

## Literature review and research hypotheses

### Sustainable development effects of environmental education

The role of education investment in sustainable development has attracted more and more attention from the academic community. Education can reduce carbon emissions, and designing a comprehensive policy framework to define sustainable development is an issue facing most countries worldwide (10). International experience shows that education and sustainable development are closely linked and that environmental education is integral to achieving sustainable development (11). Environmental education has an impact on environmental quality (12, 13).

Some studies have found that educational institutions are increasingly important in driving sustainable development. Nomura (14) studied the historical development of environmental education in Indonesia and found that education for sustainable development plays an important role in social politics, cultural freedom, and poverty eradication. Zafar et al. (10) empirically verified that the environmental quality of a region is significantly affected by the education level of residents. Getting higher education can help students rethink the role of human beings on the planet to deal with significant challenges related to sustainability (15). Azeiteiro et al. (16) surveyed 1,257 students from Portuguese public higher education institutions *via* an online questionnaire. They found that most students with education in sustainable development are more concerned about climate change and are more inclined to reuse, reduce and recycle waste. Another group of students contributes to sustainable development by participating in activities organized by environmental organizations or higher education institutions, such as environmental protection or community volunteering. Based on Australian higher education data from 1950 to 2014, Balaguer and Cantavella (6) found that the increase in per capita income and education level has been proven to be effective in reducing pollution emissions. Therefore, we propose the following:

*H1: Environmental education has a positive impact on environmental quality*.

### Influence mechanism of environmental education

Many scholars have analyzed the transmission mechanism of environmental education to sustainable development from green consumption and pollution control perspectives. Firstly, from the standpoint of consumption, relevant scholars have found that environmental education can enhance their awareness of green consumption. Publicity and education remain the primary interventions for promoting green consumption (17). Environmental education can impact consumers' willingness to consume green products by influencing consumers' environmental ethics, moral obligations, and environmental attitudes (7). Jin and Li (18) found that environmental education can effectively increase the incentives, willingness, and amount of individuals to pay for pro-environmental behaviors. Hoffman and Muttarak (19) found an association between education and pro-environmental behavior, and that education influences behavior mainly by raising awareness of anthropogenic causes of climate change. Meyer (20) found that education makes individuals pay more attention to social welfare and act more environmentally friendly by employing European data samples. Hoang (21) took Da Nang City, Vietnam, as an example, studied the impact of environmental education at the elementary level on sustainable development, and found that environmental education has significantly increased students' knowledge of solid waste management and improved environmental awareness. Educational institutions positively exported environmental protection and sustainable development courses to trainees (22).

Secondly, from the perspective of production, relevant scholars have found that environmental education can enhance enterprises' awareness of green production. The mainstream view is that pollution control can improve environmental quality, and green innovation and energy investment positively affect environmental quality. Based on Chinese provincial data, Guo et al. (23) found that income, environmental innovation, investment in the energy industry, and renewable energy consumption were critical factors in explaining CO2 emissions. Corporate innovation has significantly increased corporate environmental investment, and the regulatory effect of environmental policy is positive and significant (24). Zhou et al. (25) demonstrated that firms with highly educated CEOs are more likely to engage in environmental innovation and green production, especially when they operate in areas with severe environmental stress. At the same time, consumer environmental awareness will encourage enterprises to increase cleaner production (25). Li and Lv (26) believed that the value of consumers' environmental awareness would significantly impact the investment in environmental innovation of monopolistic manufacturers. The environmental protection industry plays a crucial role in the green economy. Fan et al. (8) analyzed the impact of investment in the environmental protection industry on the national economy from the perspective of input-output. They found that the environmental protection industry can effectively promote the national economy and employment and drive the development of other industries, especially manufacturing. King (27) even proposed that the aid of the United Nations and international organizations to backward countries and regions should not be limited to material aid but should popularize education to ensure sustainable economic development.

Thus, we believe that the impact of environmental education on environmental quality is mainly based on two channels: upgrading residents' consumption and increasing pollution control. The following hypotheses are proposed:

*H2a: Residents' consumption level will affect the quality of the environment, so it is necessary to improve the promoting effect of environmental education on environmental quality by improving residents' environmental awareness or upgrading green consumption*.
*H2b: Green technology innovation will affect environmental quality, so it is necessary to improve the promoting effect of environmental education on environmental quality by improving the cleaner production capacity of enterprises or pollution control*.

### Synergistic mechanism of environmental education and pollution control

The current views also hold that whether environmental education can improve environmental quality is still uncertain. Powdthavee (9) found that there is no evidence that changes in the minimum school age in England and Wales significantly impact individuals' environmental tendencies; that is, increasing the number of years of general education has no significant impact on people's environmental awareness. It is different whether pollution control is a pre-emptive action or a remedial measure that affects environmental quality. When pollution control is remedial, the control effect is insignificant. Because pollution problems are discovered, more environmental issues have become icebergs hidden under the sea. If pollution control is a preemptive action, the improvement in environmental quality is noticeable. On the whole, investment in environmental education indirectly impacts the progress of environmental quality, which has become the academic consensus; environmental education will affect pro-environmental behavior, but not directly. It is to improve environmental quality in an indirect way, such as knowledge-concern-willingness (28).

Li et al. (1) found that green innovation, clean energy investment, and education improve environmental sustainability in the long run. Still, short-term estimates vary and suggest governments in highly polluting economies should increase investment in education, clean energy, and technology. Haque and Sharif (29) believed that relying solely on a large number of environmental protection legislation and investment plans for environmental management has no significant effect on reducing the increasing risk of environmental pollution in Bangladesh. Environmental education needs to be strengthened. Thus, we propose the following:

*H3: It is not obvious to improve environmental quality only by relying on pollution control, which needs the support of environmental education, especially in highly polluted areas*.

## Theoretical model

Investment in environmental education can be regarded as an input element of production. The products are used for current consumption, investment in expanded reproduction in the next period, and investment in environmental quality in the next period. The investment accumulation will not be used for the next production period. From the consideration of the central planner, we draw on the Ramsey Model (30) and construct the following model to maximize the utility of consumers in each period:

(1) $$\begin{array}{l} \begin{array}{l} \text{Max}\sum_{\text{t}=0}^{\infty }\beta^{t}\ln\;c_{t}\\ \text{s}.\text{t}.\;k_{0},e_{0}\;given\;c_{t}+k_{t+1}+e_{t+1}={\left(Ak_{t}^{\alpha }e_{t}^{\tau }\right)}^{\gamma } \end{array} \end{array}$$

Where β is the intertemporal discount factor of consumption, β ∈ (0, 1). *e* represents the investment of environmental governance. *c* is the consumption. *k* is the investment. *A* is the technical efficiency, which is exogenous. α is the output elasticity of capital, α ∈ (0, 1). τ is the output elasticity of environmental investment, τ ∈ (0, 1). γ is the coordination between production and consumption, γ ∈ [0, 1]. When the consumption structure is upgraded, the production or industrial structure is also upgraded and γ is larger. Thus, we assume that $k_{t+1}=\delta k_{t}^{\alpha \gamma }e_{t}^{\tau \gamma }$,$e_{t+1}=\vartheta k_{t}^{\alpha \gamma }e_{t}^{\tau \gamma }$.

We construct the Bellman Equation:

(2) $$\begin{array}{l} V\left(k_{t},e_{t}\right){=}_{c_{t}k_{t+1}}^{max}\left\{\ln\;c_{t}+\beta V\left(k_{t+1},e_{t+1}\right)\right\} \end{array}$$

The simplified Bellman equation is as follows:

(3) $$\begin{array}{l} V\left(k_{t},e_{t}\right){=}_{k_{t+1},e_{t+1}}^{max}\left\{\ln\left(A^{\gamma }k_{t}^{\alpha \gamma }e_{t}^{\tau \gamma }-k_{t+1}-e_{t+1}\right)+\beta V\left(k_{t+1},e_{t+1}\right)\right\} \end{array}$$

From formula (3), we derive first-order derivation of *k*_*t*+1_ and *k*_*t*_ in Bellman Equation:

(4) $$\begin{array}{l} k_{t+1}:\;\frac{1}{A^{\gamma }k_{t}^{\alpha \gamma }e_{t}^{\tau \gamma }-k_{t+1}-e_{t+1}}=\;\beta V_{1}\left(k_{t+1},e_{t+1}\right) \end{array}$$

(5) $$\begin{array}{l} k_{t}:\;V_{1}\left(k_{t},e_{t}\right)=\frac{A^{\gamma }\alpha \gamma k_{t}^{\alpha \gamma -1}e_{t}^{\tau \gamma }}{A^{\gamma }k_{t}^{\alpha \gamma }e_{t}^{\tau \gamma }-k_{t+1}-e_{t+1}} \end{array}$$

Bringing $k_{t+1}=\delta k_{t}^{\alpha \gamma }e_{t}^{\tau \gamma }$,$e_{t+1}=\vartheta k_{t}^{\alpha \gamma }e_{t}^{\tau \gamma }$ into formulas (4) and (5), we have:

(6) $$\begin{array}{l} \delta =A^{\gamma }\beta \alpha \gamma \end{array}$$

Similarly, we derive first-order derivation of *e*_*t*+1_ and *e*_*t*_ in Bellman Equation from formula (3):

(7) $$\begin{array}{l} e_{t+1}:\;\frac{1}{A^{\gamma }k_{t}^{\alpha \gamma }e_{t}^{\tau \gamma }-k_{t+1}-e_{t+1}}=\;\beta V_{2}\left(k_{t+1},e_{t+1}\right) \end{array}$$

(8) $$\begin{array}{l} e_{t}:\;V_{2}\left(k_{t},e_{t}\right)=\frac{A^{\gamma }\tau \gamma k_{t}^{\alpha \gamma }e_{t}^{\tau \gamma -1}}{A^{\gamma }k_{t}^{\alpha \gamma }e_{t}^{\tau \gamma }-k_{t+1}-e_{t+1}} \end{array}$$

Bringing $k_{t+1}=\delta k_{t}^{\alpha \gamma }e_{t}^{\tau \gamma },e_{t+1}=\vartheta k_{t}^{\alpha \gamma }e_{t}^{\tau \gamma }$ into formulas (7) and (8), we have:

(9) $$\begin{array}{l} \vartheta ={\text{A}}^{\gamma }\beta \tau \gamma \end{array}$$

Therefore, in the equilibrium path, we have the following:

(10) $$\begin{array}{l} {\text{k}}_{\text{t}+1}={\text{A}}^{\gamma }\beta \alpha \gamma {\text{k}}_{\text{t}}^{\alpha \gamma }{\text{e}}_{\text{t}}^{\tau \gamma } \end{array}$$

(11) $$\begin{array}{l} {\text{e}}_{\text{t}+1}={\text{A}}^{\gamma }\beta \tau \gamma {\text{k}}_{\text{t}}^{\alpha \gamma }{\text{e}}_{\text{t}}^{\tau \gamma }\; \end{array}$$

(12) $$\begin{array}{l} {\text{c}}_{\text{t}+1}=\left(1-\beta \alpha \gamma -\beta \tau \gamma \right){\text{A}}^{\gamma }{\text{k}}_{\text{t}}^{\alpha \gamma }{\text{e}}_{\text{t}}^{\tau \gamma } \end{array}$$

Formula (10)–(12) show that with increased investment in education, investment in environmental governance will also increase. This is conducive to raising the level of capital accumulation, which in turn promotes economic growth. Correspondingly, economic growth will lead to increased investment in environmental education. The increase in investment in environmental education will not only activate citizens' awareness of environmental protection (6, 20, 21) and the rule of law (29) but also urge the government and enterprise departments to pay more attention to environmental regulation and the supply of ecological and green products (23, 26).

Formulas (10)–(12) also show that the performance of environmental education investment is highly correlated with the coordination degree of production and consumption; When the product (or industrial) structure upgrading is more consistent with the consumption structure upgrading, that is, when the value of γ is larger, the investment in environmental education is more conducive to the upgrading of household consumption, economic growth, and the improvement of environmental quality.

**Figure 1:**
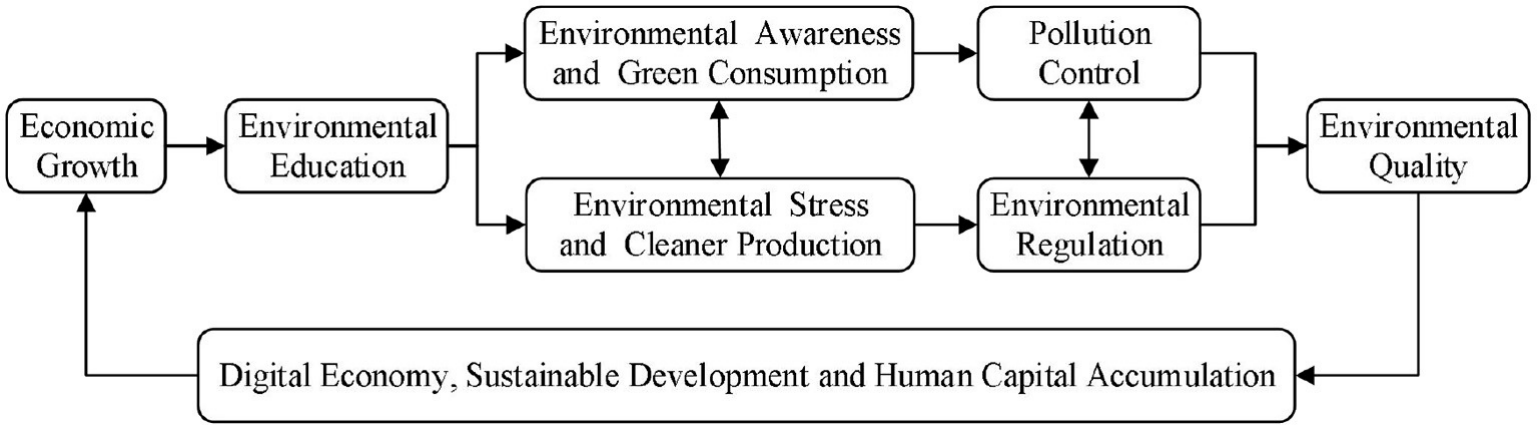
Economic growth and environmental sustainability.

Figure 1 shows that economic growth drives the improvement of environmental education. Environmental education raises residents' awareness of environmental protection. Residents resisted pollution and forced the government to implement environmental legislation and control environmental pollutants. At the same time, as consumption upgrades, consumption has become more and more prominent in green consumption and ecological consumption, which promotes green production ecological production, which is conducive to reducing pollutant emissions during production and improving environmental quality. The improvement of environmental quality provides a good atmosphere for production and consumption. It is conducive to accumulating human capital, which acts on the production process and promotes economic growth. Correspondingly, the improved environmental quality and the measures to promote it are conducive to transforming from a traditional economy to a digital economy and enterprises' adherence to low-carbon and sustainable development. Both digital economy and sustainable development are conducive to the endogenous growth of the economy through the accumulation of human capital, which is also a higher form of economic growth mode, that is, to realize the upgrading and transformation of economic development mode.

## Empirical analysis

### Variables

#### Dependent variable

Comprehensive Pollution Index (*pl*) serves as the explanatory variable in this study. Existing studies have mainly used single pollutant emission indicators as a substitute for environmental pollution, such as nitrogen oxides and sulfur dioxide (31). Industrial solid waste, industrial wastewater, soot, and domestic garbage are important factors affecting environmental quality (32–35). Entropy weight method can effectively investigate the change degree of each index and objectively reflect its importance. It can construct a pollution index with six secondary indexes, including nitrogen oxide, sulfur dioxide, industrial solid waste, industrial wastewater, soot, and household waste. The specific calculation method is as follows:

Step 1: Normalize pollution indicators.
  (13) $$\begin{array}{l} y_{ij}=\frac{x_{ij}-\min\left(x_{1j}\cdots x_{nj}\right)}{\max\left(x_{1j}\cdots x_{nj}\right)-\min\left(x_{1j}\cdots x_{nj}\right)} \end{array}$$
  Where *x*_*ij*_ is the *i*-th sample under the *j-*th pollution index, and *y*_*ij*_ is the normalized sample.
Step 2: We calculate the weight of *y*_*ij*_.
  (14) $$\begin{array}{l} p_{ij}=y_{ij}/\sum_{i=1}^{n}y_{ij} \end{array}$$
Step 3: We calculate the entropy value of the pollution index.
  (15) $$\begin{array}{l} e_{j}=-\frac{\sum_{i=1}^{n}p_{ij}\ln\;\left(p_{ij}\right)}{\ln\;\left(n\right)} \end{array}$$
  If *p*_*ij*_ = 0, then *p*_*ij*_ln(*p*_*ij*_) = 0.
Step 4: We calculate the information utility value of each pollution index.
  (16) $$\begin{array}{l} d_{j}=1-e_{j} \end{array}$$
Step 5: We calculate the weight of each pollution index.
  (17) $$\begin{array}{l} w_{j}=\frac{d_{j}}{\sum_{j=1}^{m}d_{j}} \end{array}$$
Step 6: We calculate the comprehensive pollution index.
  (18) $$\begin{array}{l} pl_{i}=\sum_{j=1}^{m}w_{j}y_{ij} \end{array}$$

#### Independent variables

Environmental education (*eedu*) is the core explanatory variable. Since environmental education is not listed in China's statistical data, we will use the fiscal expenditure for education as an alternative indicator of environmental education. The choice of proxy variable is based on two aspects. First, the Ministry of Education and the Ministry of Environmental Protection of the People's Republic of China jointly formulated the National Measures for the Application and Management of Social Practice Bases of Environmental Education in Primary and Secondary Schools, which is committed to standardizing the construction and management of social practice bases of environmental education in primary and secondary schools. Second, the Ministry of Education of the People's Republic of China issued the Notice on Implementing the Xi Jinping Thought of Ecological Civilization and Enhancing the Awareness of Ecological Environment in Primary and Secondary schools, requiring educational administrative departments at all levels and primary and secondary schools to reflect entirely thrives, green and low-carbon consumption in the teaching of relevant subjects, in-class and extra-curricular activities and all links of school management, so that students can effectively enhance awareness of the ecological environment. Improve the ability to protect the ecological environment. This means that all kinds of schools relying on fiscal education expenditure will carry environmental education through the whole education process; that is, education expenditure is an appropriate proxy variable for environmental education expenditure.

In the robustness test, we use the number of environmental publicity and education (*nedu*) as a substitute indicator of environmental education. In the theoretical analysis, we find that environmental education will affect the environmental pollution index through residential consumption and pollution control. So other independent variables are resident consumption (*rc*) and investment in pollution control (*pci*). The level of household consumption expresses resident consumption, and pollution control is measured by the amount of investment completed in industrial pollution control.

#### Other variables

Other variables conclude total population (*tp*), the proportion of the secondary industry (*psi*), amount of electricity consumption (*aec*), the degree of openness (*open*), and the fiscal and taxation capabilities (*ftc*). The global environmental change caused by humans has accelerated unprecedentedly, and the continued population growth has a particular relationship with environmental degradation (36). The upgrading of industrial structure accompanies economic growth, and the impact of industrial development on environmental quality is more obvious (37). At the same time, power consumption is an important manifestation of economic activity, and there have been studies using city lights to reflect economic development (38). It can be considered that secondary industry and power consumption are important factors affecting environmental quality. The division of labor and cooperation in international trade has brought environmental pollution problems, such as overseas investment in high-polluting industries (39). A country's tax revenue affects the ability to control pollution and the investment in environmental education. Therefore, when analyzing the impact of environmental quality, we consider a country's degree of openness and tax revenue.

Table 1 shows the definition of the variables. In the regression analysis, we take the logarithm of the variables (*eedu, nedu, rc, pci, tp*, and *aec*). The data in this article comes from the “China Statistical Yearbook” and “China Environmental Statistical Yearbook” from 2011 to 2017. Table 2 shows the statistical description of the variables.

**Table 1:** Definition of the variables.

**Variables**			**Description**
Dependent variable	*pl*		Calculated from six pollution indicators using entropy method
	Secondary indicators	Nitrogen oxides	Nitrogen oxide emissions per unit of GDP
		Sulfur dioxide	Sulfur dioxide emissions per unit of GDP
		Industrial solid waste	Industrial solid waste discharged per unit of GDP
		Industrial wastewater	Wastewater discharged per unit of GDP
		Dust	Smoke and dust generated per unit of GDP
		Domestic garbage	Domestic waste generated per unit of GDP
Independent variables	*eedu*		Financial expenditure for education
	*nedu*		Number of environmental publicity and education
	*rc*		Resident consumption level
	*pci*		Complete investment in the treatment of industrial pollution
Other variables	*tp*		Total population
	*psi*		The proportion of the secondary industry
	*aec*		Amount of electricity consumption
	*Open*		Total import and export/GDP
	*ftc*		Tax revenue/GDP

**Table 2:** The statistical description of the variables.

**Variables**	**Observations**	**Mean**	**Median**	**Max**	**Min**
*pl*	217	0.191	0.151	0.720	0.017
*eedu*	217	6.396	6.475	7.854	4.354
*nedu*	155	5.593	5.505	8.731	2.890
*rc*	217	9.685	9.629	10.890	8.462
*pci*	217	2.682	2.856	4.953	−2.668
*tp*	217	8.122	8.244	9.404	5.733
*psi*	217	0.451	0.472	0.590	0.190
*aec*	217	7.176	7.252	8.693	3.168
*Open*	217	0.043	0.022	0.240	0.002
*ftc*	217	0.085	0.079	0.200	0.047

### Empirical model

Based on theoretical analysis and research hypotheses, the baseline model is set as follows:

(19) $$\begin{array}{l} pl_{it}=\beta_{0}+\beta_{1}rc_{it}+\beta_{2}pci_{it}+\beta_{3}eedu_{it}+control+year\\ \;+province+\varepsilon_{it} \end{array}$$

(20) $$\begin{array}{l} pl_{it}=\beta_{0}+\beta_{1}rc_{it}+\beta_{2}pci_{it}+\beta_{3}eedu_{it}+\beta_{4}eedu\times rc_{it}\\ \;+control+year+province+\varepsilon_{it} \end{array}$$

(21) $$\begin{array}{l} pl_{it}=\beta_{0}+\beta_{1}rc_{it}+\beta_{2}pci_{it}+\beta_{3}eedu_{it}+\beta_{4}eedu\times pci_{it}\\ \;+control+year+province+\varepsilon_{it} \end{array}$$

Where *i* and *t* represent province and year, respectively. *pl* stands for pollution index, *rc* stands for household consumption, *pci* stands for pollution control investment, and *eedu* stands for environmental education investment. *control* contains other variables (*tp, psi, aec, open, ftc*). *year* represents the time effect of the province, and *province* represents the individual fixed effect of the province. ε denotes random disturbance terms.

### Baseline regression results

#### Correlation coefficient test

The correlation coefficients between variables and their significance levels are shown in Table 3. Although the correlation between *pl* and *pci* is not statistically significant, *pl* is negatively correlated with *eedu, rc*, and *pci*. *eedu* has a significant positive correlation with *rc* and *pci*. Population, electricity consumption, economic openness, and tax revenue negatively correlate with the environmental pollution index. The development of the secondary industry has aggravated environmental pollution. Initially, the investment in environmental education is conducive to environmental quality, and residents' consumption and pollution control are also conducive to environmental quality. Environmental education positively impacts residents' consumption upgrades and pollution control. Environmental education may affect environmental quality through two channels, influencing residents' consumption upgrade and pollution control. Of course, the conclusion is that the influence of other factors has not been considered. Next, we consider other influencing factors for further empirical verification.

**Table 3:** Correlation coefficients of variables.

	***pl***	***eedu***	***rc***	***pci***	***tp***	***psi***	***aec***	***open***	***ftc***
*pl*	1								
*eedu*	−0.521^***^	1							
*rc*	−0.518^***^	0.449^***^	1						
*pci*	−0.061	0.661^***^	0.338^***^	1					
*tp*	−0.346^***^	0.912^***^	0.179^***^	0.704^***^	1				
*psi*	0.324^***^	0.105	−0.318^***^	0.359^***^	0.299^***^	1			
*aec*	−0.094	0.808^***^	0.428^***^	0.855^***^	0.828^***^	0.343^***^	1		
*Open*	−0.437^***^	0.259^***^	0.658^***^	0.037	0.081	−0.352^***^	0.169^**^	1	
*ftc*	−0.184^***^	−0.087	0.507^***^	−0.196^***^	−0.302^***^	−0.627^***^	−0.170^**^	0.730^**^	1

(1) ^***^p < 0.01; ^**^p < 0.05; ^*^p < 0.1. (2) Standard errors are in parentheses.

#### Baseline regression results

Table 4 shows the baseline regression results. Model (1) shows that environmental education, household consumption levels, and pollution control significantly negatively impact the pollution index. The improvement of environmental education, the increase of residents' consumption level, and the increase of pollution control are all conducive to environmental quality. The result verifies *H1*.

**Table 4:** Baseline regression results.

**Variables**	**(1)**	**(2)**	**(3)**
*eedu*	−0.089^***^	−0.285^*^	−0.044
	(0.027)	(0.146)	(0.033)
*rc*	−0.233^***^	−0.348^***^	−0.246^***^
	(0.024)	(0.088)	(0.025)
*pci*	−0.016^**^	−0.018^**^	0.047^*^
	(0.008)	(0.008)	(0.028)
*eedu* × *rc*		0.019	
		(0.014)	
*eedu* × *pci*			−0.010^**^
			(0.004)
*tp*	−0.136^***^	−0.128^***^	−0.153^***^
	(0.023)	(0.024)	(0.024)
*psi*	0.060	0.068	0.057
	(0.085)	(0.085)	(0.084)
*aec*	0.217^***^	0.220^***^	0.213^***^
	(0.013)	(0.013)	(0.013)
*Open*	−0.664^***^	−0.737^***^	−0.559^***^
	(0.208)	(0.215)	(0.211)
*ftc*	1.427^***^	1.485^***^	1.275^***^
	(0.290)	(0.293)	(0.294)
Constant	2.490^***^	3.569^***^	2.509^***^
	(0.232)	(0.824)	(0.229)
Year fixed effect	YES	YES	YES
Province fixed effect	YES	YES	YES
*N*	217	217	217
Adjusted *R*^2^	0.820	0.820	0.824
$\bar{eedu}$	−0.089	−0.101	−0.071

(1) ^***^p < 0.01; ^**^p < 0.05; ^*^p < 0.1.

(2) Standard errors are in parentheses.

Model (2) shows that the interaction between environmental education and residents' consumption level is positive (*eedu* × *rc* = 0.019) but insignificant. It does not indicate that the impact of environmental education on environmental quality will decrease as the level of resident consumption increases. Meanwhile, the coefficients of environmental education, resident consumption, and pollution control are all significantly negative, indicating that the three have a promoting effect on improving environmental quality. The impact of environmental education and pollution control on environmental quality is consistent with the existing research conclusions. The reason resident consumption can improve environmental quality may be that with the deepening of environmental education, resident consumption pays more and more attention to green consumption (7), and green consumption can reduce the degree of environmental pollution (40). The conclusion verifies the *H2a*.

The influence coefficient of environmental education on environmental quality can also be verified by calculating the partial effect of environmental education. The calculation formula of the partial effect of environmental education incorporated into the resident consumption level is as follows: $\bar{eedu}=eedu+eedu\times \text{Mean}\left(rc\right)$. The results show that environmental education has a negative impact on the pollution index (−0.101); that is, environmental education investment has improved environmental quality. The conclusion still verifies the *H1*.

Model (3) shows that the interaction between environmental education and pollution control (*eedu* × *pci*) is significantly negative. That is, with the increase of pollution control efforts, the impact of environmental education on environmental quality gradually increases. The conclusion verifies the *H2b*. It is worth noting that the coefficient of pollution control on environmental quality is significantly positive, which means that the investment in environmental control will increase the pollution level. The possible reason is that pollution control is relatively passive; that is, areas with more investment in pollution control often have more severe pollution. In Section 4.5 of this paper, quantile regression will be conducted according to the regional pollution degree to explore the reasons for this phenomenon further. In consideration of the coefficient value of *eedu* is not significantly negative. Similarly, we use Formula ($\bar{eedu}=eedu+eedu\times \text{Mean}\left(pci\right)$) to solve the partial effect of environmental education incorporating pollution control. The partial effect of environmental education ($\bar{eedu}$) is −0.071; environmental education investment improves environmental quality. The conclusion still verifies the *H1*. At the same time, this partial effect examines the main effect of environmental education and the interaction effect of pollution control and environmental control, so a smaller negative partial effect coefficient ($\bar{eedu}$ = −0.071, *eedu* = −0.044) can also support *H2b*.

In addition, there is a significant negative correlation between the region's total population and the environmental pollution index. It can be seen that the larger the number of people, the more attention is paid to the environment, especially in large cities. Although the secondary industry positively correlates with the environmental pollution index, it is not statistically significant. However, there is a significant positive correlation between regional power consumption and the environmental pollution index, which to a certain extent, reflects that China's economic development has brought certain environmental pollution problems. There is a positive correlation between tax revenue and the environmental pollution index. The possible reason is that economic development sacrifices the environment, and tax revenue depends on economic growth. Increased taxation does not necessarily lead to a significant increase in investment in environmental pollution control. Table 4 shows a significant negative correlation between tax revenues and investment in environmental governance. Tax revenue has a negative correlation with environmental education.

### Robust regression results

Table 5 shows the robustness regression results of this article. We use *nedu* as the key independent variable. Model (4) shows that environmental education, residents' consumption level, and pollution control significantly negatively impact the pollution index. Environmental education, residents' consumption level, and pollution control are all conducive to environmental quality. The robustness test verifies *H1*.

**Table 5:** Robust regression results by replacing the key independent variable.

**Variables**	**(4)**	**(5)**	**(6)**
*nedu*	−0.015^**^	−0.358^**^	0.037^**^
	(0.006)	(0.158)	(0.016)
*rc*	−0.278^***^	−0.472^***^	−0.293^***^
	(0.032)	(0.094)	(0.031)
*pci*	−0.029^***^	−0.032^***^	0.082^**^
	(0.011)	(0.011)	(0.034)
*nedu* × *rc*		0.036^**^	
		(0.016)	
*nedu* × *pci*			−0.019^***^
			(0.006)
Constant	2.874^***^	4.709^***^	2.826^***^
	(0.289)	(0.888)	(0.279)
Control	YES	YES	YES
Year fixed effect	YES	YES	YES
Province fixed effect	YES	YES	YES
*N*	155	155	155
Adjusted *R*^2^	0.827	0.832	0.840
$\bar{nedu}$	−0.015	−0.009	−0.014

(1) ^***^p < 0.01; ^**^p < 0.05; ^*^p < 0.1.

(2) Standard errors are in parentheses.

In model (5), The inhibitory effect of environmental education on environmental pollution is significantly negative (−0.358), but the interaction between environmental education and residents' consumption level (*nedu* × *rc*) is significantly positive. It means that with the increase in residents' consumption, the inhibitory effect of environmental education on environmental pollution will gradually weaken. This makes it even more necessary to improve consumers' awareness of green consumption and achieve environmental protection through green consumption. The conclusion verifies the *H2a*. Consistent with the benchmark regression, the influence coefficient of environmental education on environmental quality can also be verified by calculating the partial effect of environmental education. The calculation formula of the partial effect of environmental education incorporated into residents' consumption level is as follows: $\bar{nedu}=nedu+nedu\times \text{Mean}\left(rc\right)$. The partial effect of environmental education impact ($\bar{nedu}$) is −0.009; that is, environmental education investment improves environmental quality. The robustness test verifies the *H1*.

In model (6), the interaction between environmental education and pollution control is considered, and it is found that *nedu* × *pci* is significantly negative. Still, the coefficient value of *nedu* is significantly positive, and the coefficient of *pci* is significantly positive. This shows that environmental education may affect the environmental level through pollution control investment. To some extent, this indicates that there is uncertainty in the direction of the effect of environmental education on environmental quality once the moderating effect of pollution control investment intensity is included. As the intensity of pollution control increases, the effect of environmental education on environmental quality shows a decreasing trend. This paradox will be solved by quantile regression. The coefficient of *nedu* is significantly positive; it is necessary to calculate the partial effect of environmental education, which is calculated as follows: $\bar{nedu}=nedu+nedu\times \text{Mean}\left(pci\right)$. The partial effect of environmental education impact ($\bar{nedu}$) is −0.014; that is, environmental education improves environmental quality. Robust regression results are consistent with baseline regression results. The robustness test verifies *H1*.

### Quantile regression results

To further analyze the results of model (3) and model (6) and verify the impact of pollution control on the environmental pollution index, we conducted quantile regression analysis according to the size of the pollution control investment. Table 6 shows environmental education and residents' consumption level significantly negatively impact the pollution index; that is, environmental education and residents' consumption level are all conducive to environmental quality. Moreover, from the quantile regression coefficient, the reduction of the pollution index is more dependent on environmental education and residents' green consumption tendency.

**Table 6:** Quantile regression results.

**Variables**	**(7)**	**(8)**	**(9)**	**(10)**	**(11)**
*eedu*	−0.076^***^	−0.096^***^	−0.097^***^	−0.129^***^	−0.078^***^
	(0.013)	(0.022)	(0.023)	(0.028)	(0.027)
*rc*	−0.170^***^	−0.209^***^	−0.225^***^	−0.202^***^	−0.274^***^
	(0.012)	(0.020)	(0.025)	(0.026)	(0.025)
*pci*	−0.022^***^	−0.020^***^	−0.014^*^	−0.006	−0.004
	(0.004)	(0.006)	(0.008)	(0.008)	(0.008)
Control	YES	YES	YES	YES	YES
Quantile	0.10	0.35	0.50	0.65	0.90

(1) ^***^p < 0.01; ^**^ *p* < 0.05; ^*^ *p* < 0.1.

(2) Standard errors are in parentheses.

It is worth noting that with the improvement of the region's pollution index, the pollution control role is getting smaller and smaller. The possible reason is that the increase in investment in pollution control is a passive choice. First, in areas with severe pollution, investment in pollution control has to be increased to meet the environmental protection requirements of the central government. Second, improving residents' environmental awareness will also encourage government departments to increase investment in environmental governance. Residents' emphasis on the ecological environment and protests against polluting companies or behaviors have forced local governments to increase their control of environmental pollution (41). Quantile regression results verify the *H3*.

## Conclusions and discussions

### Conclusions

The low-carbon economy is the dominant choice for human society to solve the dilemma of environmental governance. The high-quality natural environment provides a good production and consumption atmosphere for economic growth. This paper's theoretical and empirical research can provide the following four conclusions. First, environmental education will significantly reduce the environmental pollution index, which can be supported by the benchmark regression coefficient of environmental education and its partial effect coefficient.

Second, from the perspective of green consumption and cleaner production channels, with the improvement of residents' consumption level, the promotion effect of environmental education on the progress of environmental quality shows a downward trend, so it is more necessary to truly enhance residents' awareness of green consumption through environmental education. It will also push companies toward cleaner production through technological innovation and environmental investment.

Third, from the perspective of pollution control channels, pollution control investment cannot effectively promote environmental quality because there is a paradox of positive correlation between regional pollution degree and pollution investment amount. In areas with higher pollution degrees, more pollution control costs need to be invested in meeting environmental protection standards or residents' willingness to protect environmental protection. The conclusion of quantile regression verifies this phenomenon.

In addition, we find that there is uncertainty about pollution control in improving environmental quality. With the improvement of the pollution index of the region itself, the role of pollution control is getting smaller and smaller. This indicates that the environmental governance issues in areas with higher pollution levels are more passive. It also means that environmental education should be carried out first to avoid passive responses when environmental pollution is severe.

### Managerial implications

First of all, it is necessary to form a consensus on environmental protection in the whole society through environmental education from a strategic perspective. Chinese local governments should respond to the unified deployment of the CPC Central Committee and the State Council of the People's Republic of China, distinguish between different objects such as state organs, enterprises, public institutions, social organizations, schools, families, and the public, and penetrate environmental education into family education, primary education, higher education, vocational education, cadre education, and training and other links. Environmental education should penetrate all stages of citizens' lives according to its purpose and tasks, combined with its universality and life-long characteristics. This is an important measure to implement the concept that clear waters and lush mountains are gold and silver mountains and also a necessary choice for a low-carbon economy and sustainable development.

Secondly, from the perspective of the channel mechanism of environmental education. Environmental education should emphasize its role in promoting citizens' environmental awareness and reflect the positive role of environmental education in promoting residents' green consumption and enterprises' green production. At the same time, local environmental protection and environmental education departments should actively respond to hot environmental issues concerned by the people with a scientific and pragmatic attitude, guide the public to participate in environmental protection work in a mature, rational, orderly, and effective manner, gather the people's hearts, wisdom and strength to the greatest extent, and jointly promote the steady improvement of environmental quality.

Finally, environmental education legislation should be accelerated from the perspective of legal regulation. Currently, the connection between environmental education and legislation in China exists only in single words and sentences of individual legal provisions. For example, the Environmental Protection Law only stipulates the principles of environmental education. According to the Environmental Protection Law of the People's Republic of China, the Education Law of the People's Republic of China and other laws and regulations, Jiangsu Province has formulated the Measures for the Promotion of Ecological Civilization Education of Jiangsu Province, which also defines the scope and implementation subjects of ecological civilization education in schools, families, and society. However, it is still only an administrative document of the local government. It has not yet risen to the level of the legal system. In fact, environmental education legislation can regulate the behavior of citizens and enterprises and provide a legal system guarantee for the government's environmental education and environmental governance.

### Theoretical contributions and research limitations

There are two marginal contributions in this paper. Firstly, this paper improves the Ramsey model and finds that the fit between consumption structure and industrial structure is an important factor for environmental education to improve environmental quality, which provides a factual basis for environmental protection practice. Secondly, this paper conducts theoretical analysis and empirical tests on the transmission mechanism of environmental education to environmental quality and finds that environmental education can improve environmental quality through pollution control and green consumption, especially for heavily polluted areas. Environmental protection and environmental education should be strengthened, especially for heavily polluted areas, which is an expansion of the research on the transmission mechanism of environmental education.

There are a few shortcomings in this paper. For example, due to data availability, this paper uses the data at the provincial level in China and does not analyze the city and county levels. This paper also fails to explore how environmental education affects citizens' environmental awareness and, thus, environmental quality. It does not explore the influencing mechanism of environmental education on high-quality economic and social development, which are future research directions.

## Data availability statement

The original contributions presented in the study are included in the article/supplementary material, further inquiries can be directed to the corresponding author.

## Author contributions

YW, JW, and WY conceived the study and drafted the manuscript. YW designed the model. All authors contributed to the article and approved the submitted version.
